# ESUR consensus MRI for endometriosis: indications, reporting, and classifications

**DOI:** 10.1007/s00330-025-11579-0

**Published:** 2025-05-27

**Authors:** Isabelle Thomassin-Naggara, Miriam Dolciami, Luciana P. Chamie, Adalgisa Guerra, Nishat Bharwani, Susan Freeman, Pascal Rousset, Lucia Manganaro, the ESUR Endometriosis Working Group, Miriam Dolciami, Luciana P. Chamie, Nishat Bharwani, Pascal Rousset, Lucia Manganaro, Adalgisa Guerra, Giacomo Avesani, Marc Bazot, Teresa Margarida Cunha, Paolo Niccolò Franco, Sue Freeman, Rosemarie Forstner, Benedetta Gui, Edith Kermarrec, Stefania Rizzo, Hilal Sahin, Shiwa Mansournia, Isabelle Thomassin-Naggara, Laura Buñesch Villalba, Ramona Woitek

**Affiliations:** 1 Imageries Radiologiques et Interventionnelles Spécialisées (IRIS), APHP Sorbonne Université Hopital Tenon, Paris, France; 2 Sorbonne Université, GRC Endometriose, GRC6-C3E Paris, France; 3 Department of Imaging and Radiation Oncology, Fondazione Policlinico Universitario Agostino Gemelli IRCCS, Rome, Italy; 4 Chamie Imagem da Mulher, Sao Paulo, SP Brazil; 5 Imaging Department of Hospital da Luz Lisboa, Lisboa, Portugal; 6 Department of Imaging, Imperial College Healthcare NHS Trust, London, UK; 7 Department of Surgery & Cancer, Imperial College London, London, UK; 8 Cambridge University Hospitals, Cambridge, UK; 9 Department of radiology, Hospices Civils de Lyon, Lyon Sud University Hospital, Lyon 1 Claude Bernard University, EMR 3738, 69495 Pierre Bénite, France; 10 Department of Radiological, Pathological and Oncological Sciences, Sapienza University of Rome, Rome, Italy; 11 Department of Radiology, Instituto Portugues de Oncologia de Lisboa Francisco Gentil, Lisboa, Portugal; 12 Department of Diagnostic Radiology, IRCCS Foundation San Gerardo dei Tintori, Monza, Italy; 13 Department of Radiology, University Hospital of Salzburg, PMU, Salzburg, Austria; 14 Imaging Institute of Southern Switzerland (IIMSI), Ente Ospedaliero Cantonale (EOC), Lugano, CH Switzerland; 15 Faculty of biomedical sciences, Università della Svizzera italiana (USI), Lugano, CH Switzerland; 16 University of Health Sciences, Izmir City Hospital, Department of Radiology, Izmir, Turkey; 17 Department of Radiology, LMU University Hospital, LMU Munich, München, Germany; 18 Hospital Clínic, Servei de Radiodiagnòstic, Barcelona, Spain; 19 Research Center for Medical Image Analysis and AI (MIAAI), Danube Private University, Krems, Austria

**Keywords:** Endometriosis, Pelvis, Magnetic resonance imaging, Consensus

## Abstract

**Objective:**

To propose an update of ESUR endometriosis guidelines to reflect advances in MRI indications, reporting, and classifications.

**Methods:**

The ESUR Research Committee appointed two chairs (I.T.N., L.M.) to supervise the development of the updated guidelines. Following literature research, a survey was delivered to 20 experts in gynecological imaging from 10 countries. Two rounds of surveys were conducted to obtain a consensus according to a Delphi process method. In this article, the results regarding MR indication, the use of standardized reports, and classifications are presented

**Results:**

Magnetic resonance imaging (MRI) is recommended when transvaginal ultrasonography is inconclusive in diagnosing endometriosis or negative, in a symptomatic patient, before surgery or interventional procedure, or after surgical treatment if symptoms persist. ESUR panelists consider the roles of an MR classification: to improve communication between radiologist and surgeon (100%, 20/20) and between the radiologist and the patient (45%, 9/20), to predict operating time if surgery is planned (70%, 14/20), to predict the length of hospital stay after surgery (40%, 8/20), and to predict postoperative complications (70%, 14/20). ESUR panelists strongly agree that using an MR classification is useful (19/20, 95%), especially the radiological score, deep-pelvic endometriosis index (dPEI). Among the ESUR expert group, 9/20 experts (45%) used or agreed to use drawings in their report to improve communication with patients.

**Conclusion:**

Standardized MR reporting is crucial and should include the use of MR classification. Drawings are considered an option, knowing that communication with the patient and surgeon is of paramount importance.

**Key Points:**

***Question*** *ESUR’s endometriosis guidelines were last published in 2017; an update is provided to reflect advances in MRI indications, reporting, and classifications.*

***Findings*** *MRI is advised for inconclusive/negative transvaginal ultrasound in symptomatic patients, before surgery, or post-treatment if symptoms persist. A structured report enhances communication with surgeons and patients.*

***Clinical relevance*** *A standardized report based on a compartmental analysis of the location of endometriotic nodules, with optional drawings, is essential for comprehensive mapping and optimal communication with both patient and surgeon.*

**Graphical Abstract:**

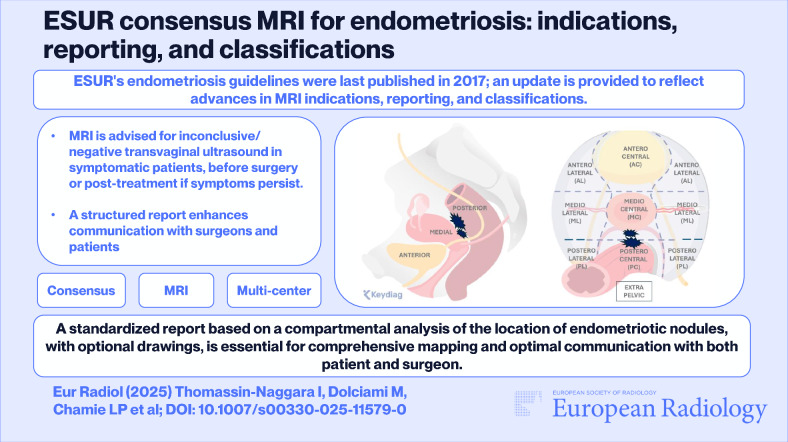

## Introduction

Magnetic resonance (MR) imaging is a second-line technique after transvaginal ultrasonography (TVUS) for exploring patients with a suspicion of endometriosis. The combination of these two imaging modalities and physical examination increases diagnostic accuracy, especially for deep-pelvic endometriosis (DE) locations [1]. Beyond its excellent sensitivity, the main advantage of MR imaging is the possibility of reviewing images by an expert or during a multidisciplinary session before deciding on surgical management [2].

As endometriosis is a benign disease that may require very complex surgery with potentially long-term impact on the quality of life of the patient [3], the standardization of MR reporting is critical. Recently, the European Society of Radiology underlined that optimal communication between the imaging team and patients is an essential component of patient-centered and value-based care [4]. This is particularly important in the context of endometriosis management. The imaging report is a crucial tool for communication.

Since the previous consensus, several authors have proposed a consensual lexicon and developed different reporting classifications to improve communication between the radiologist and the patient or the radiologist and the surgeon [5–9]. This is why it is time to change imaging guidelines in endometriosis [10].

## Methods

### Expert panel members

The panel included 20 experts from 10 different countries, including 14 European centers and one non-European institution (Italy (*n* = 5), France (*n* = 4), Austria (*n* = 2), Portugal (*n* = 2), United Kingdom (*n* = 2), Germany (*n* = 1), Spain (*n* = 1), Turkey (*n* = 1), Switzerland (*n* = 1) and Brazil (*n* = 1)). Ninety percent of panelists (18/20) were affiliated with referral centers for endometriosis with a high volume of dedicated TVUS (> 30 TVUS per month; 80%, 16/20). In half of these centers, the radiologists themselves conduct the ultrasound examinations. Regarding MR practice, more than 30 exams are acquired monthly in 50% of centers. Additionally, 50% of panelists (10/20) regularly participate in multidisciplinary meetings dedicated to endometriosis.

### Delphi process

The ESUR Research Committee appointed two chairs (I.T.N., L.M.) to supervise the development of the updated guidelines. The updating process comprised the following steps:

**Step 1**—Literature search: Four authors (I.T.N., L.M., M.D., and P.R.) performed a literature review of new data covering the time frame between 2017 and 2023 by searching PubMed/Medline database, EMBASE, and Cochrane Library for original articles in the English language on human subjects, including the following keywords: “endometriosis” AND “MRI” OR “Magnetic Resonance”.

**Step 2**—Panel selection: The chairpersons selected 20 ESUR Female Pelvic Imaging Working Group (FPI-ESUR) members based on their competence in the field; the criteria for inclusion in the group were radiologists with at least five years of experience in endometriosis imaging and who had produced relevant indexed and peer-reviewed publications.

**Step 3**—Template development: The two chairs devised draft Delphi questionnaires, which the guideline committee further improved and approved. A final questionnaire was developed with 88 questions focusing on the following topics: characteristics of the examined imaging centers, indications for MRI examination, patient preparation, MRI protocol and technical details, analysis of MRI results, MRI lexicon, and reporting.

**Step 4**—Survey delivery: In November 2023, the questionnaire was distributed electronically to all panel members before the first meeting, and responses were recorded.

**Step 5**—Data extraction and analysis: The survey responses were collected in February 2024 and analyzed; each item was classified as follows: “RECOMMENDED” (if agreement ≥ 80%); “OPTIONAL” (if agreement ≥ 70% but < 80%); or “NOT RECOMMENDED” (if consensus was not reached, with < 70% agreement). The results were presented and discussed during a virtual meeting in April 2024.

**Step 6**—Second survey: A new questionnaire version was emailed to all panel members to clarify any potentially conflicting answers that arose during the first survey round and the first meeting, and the new responses were recorded.

**Step 7**—Second and final meeting: The panel members met again in June 2024 to discuss the remaining open questions on the various items. The focus was on the questions that created debate among the experts at the first meeting.

**Step 8**—Data reporting: The final survey responses were collected and analyzed in June 2024. Six authors (I.T.N., L.M., M.D., P.R., A.G., and L.C.) then prepared the first draft of the new recommendations, which also included the statements derived from the results obtained.

The first draft was shared with the group in August 2024 for possible suggestions or improvements. The final version of the recommendation paper was proposed in September 2024.

### Determining consensus

There is no accepted, set standard for the target percentage of agreement [11], and while 70% (summative of agree (≥ 70%) and strongly agree (≥ 80%) at the second round) is commonly reported in the literature, given the importance of promoting appropriate recommendations, the consensus was deemed to have been met at 80% (summative of agree and strongly agree) for each individual statement. If the percentage obtained at the second round was lower than 70%, the term disagree was considered.

The group was also asked to grade the level of evidence using the GRADE system, following the same model as previous guidelines, with the addition of publications between 2017 and 2024 [12].

### MR indication

In previous ESUR guidelines [13], the authors identified staging DE as the main indication for MRI and cited two metanalyses that demonstrated MRI as a second-line technique in the investigation of a symptomatic patient in the presence of negative US findings [14, 15] (LE1) and a third metanalysis that demonstrated that MRI is a useful preoperative test for predicting the diagnosis of multiple sites of DE [16] (LE1). This previous consensus stated that no consensus exists in the literature regarding the use of MRI in comparison to US. Recently, a large European consensus, including 6 gynecological and one radiological society, was published comparing the value of US and MRI [2]. In this consensus, transvaginal ultrasonography (TVUS) is recommended as a first-line imaging tool, due to its availability, good test performance, cost efficacy, and low environmental impact. However, the authors also underlined that many centers adopt MRI as a first-line technique and considered that it could also be appropriate in some specific contexts. There was strong agreement that the TVUS assessment of patients with suspected DE will accurately determine or rule out the presence of DE affecting the rectum, rectovaginal septum (RVS), and bladder (LE1). However, the consensus stated that TVUS is less precise for parametrial and uterosacral ligament (USL) DE, while MRI can reliably predict all locations, including the presence of USL and parametrial DE (LE1). In line with this consensus, the current ESUR expert panel strongly agreed with the following recommended indications for MRI:

- When TVUS is inconclusive in diagnosing endometriosis (95%, 19/20).
- When TVUS is negative, but the patient is symptomatic (95%, 19/20).
- For patients who are candidates for surgical or interventional radiological treatment for endometriosis (90%, 18/20).
- After surgical treatment, if symptoms persist (90%, 18/20).

The experts did not reach an agreement regarding the use of MRI in follow-up to monitor medical treatment response (50%, 10/20), even though this type of indication is becoming more established in clinical practice.

**Statement 1:** MRI is recommended when TVUS is inconclusive in diagnosing endometriosis or negative, in a symptomatic patient (Grade A, strong agreement), before surgery or interventional procedure, or after surgical treatment if symptoms persist (strong agreement).

When TVUS is not feasible (virgins, adolescents, and patients who are unable to undergo the transvaginal examination due to pain, vaginismus, or claustrophobia), MRI can be indicated as a first-line technique.

### Classification and reporting

#### Usefulness of MR classifications

Disease staging is crucial for both clinical and research activities related to endometriosis. No single staging system is universally competent or applicable to all aspects of endometriosis care, and different approaches should be viewed as complementary. Multiple surgical and radiological classifications exist; however, a broad international consensus has not yet been reached to indicate which of these systems should be recommended for routine use.

Surgical classifications provide a comprehensive staging of DE and evaluate peritoneal, ovarian, and tubal involvement, as well as the presence of adenomyosis. Prominent among these are the American Association of Gynecologic Laparoscopists (AAGL), the revised American Society for Reproductive Medicine (rASRM), and the #Enzian classification. It is essential to recognize the distinction between the surgeon’s operative perspective and the radiologist’s imaging perspective in evaluating a given patient. The radiologist visualizes the entire pelvis in cross-section, encompassing all organs and anatomical spaces within a single visual field, in fact, in multiple anatomical orthogonal planes, which is the major advantage. In contrast, the surgeon, through laparoscopy, views the peritoneum and the external surface of underlying organs. Laparoscopic surgical classifications do not provide a comprehensive evaluation of pelvic endometriosis. The surgeon cannot initially visualize the internal structures of organs or retroperitoneal spaces, nor can they directly assess the depth of DE within organs or discern the boundaries between organs in cases of obliterated cul-de-sac or the precise location of DE lesions. Radiologists, therefore, must invest time and effort into understanding evolving disease concepts and treatment options and maintain effective communication with referring colleagues to ensure their knowledge remains current across medical fields beyond radiology. By integrating imaging expertise with an understanding of disease management, radiologists contribute significantly to patient care [17].

Among the surgical scores, the #Enzian classification has been the most studied in MRI and is the most commonly used, as it is applicable to various aspects of MRI evaluation of endometriosis [7, 18]. However, it is based on the operative view and notably fails to assess the extent of lateral pelvic wall involvement. The #Enzian classification accurately assesses A (rectovaginal space, vagina, retrocervical area) and C (rectum) lesions, but it exhibits poor reproducibility and is complex to use, particularly showing low concordance for B (USLs, cardinal ligaments, pelvic side wall) lesions. Furthermore, discrepancies exist in evaluating tubo-ovarian conditions, which are not fully assessable by MRI. Additionally, the subdivision of adhesions into three distinct groups, based on sites and involved organs, remains complex and challenging to reproduce. Another limitation is the low sensitivity of MRI in diagnosing superficial endometriosis. Regarding the reproducibility of the #Enzian classification when applied to MRI, a moderate inter-reader agreement was demonstrated for DE, indicating that accurate assessment of DE is dependent on the reader’s expertise [7].

**Fig. 1:**
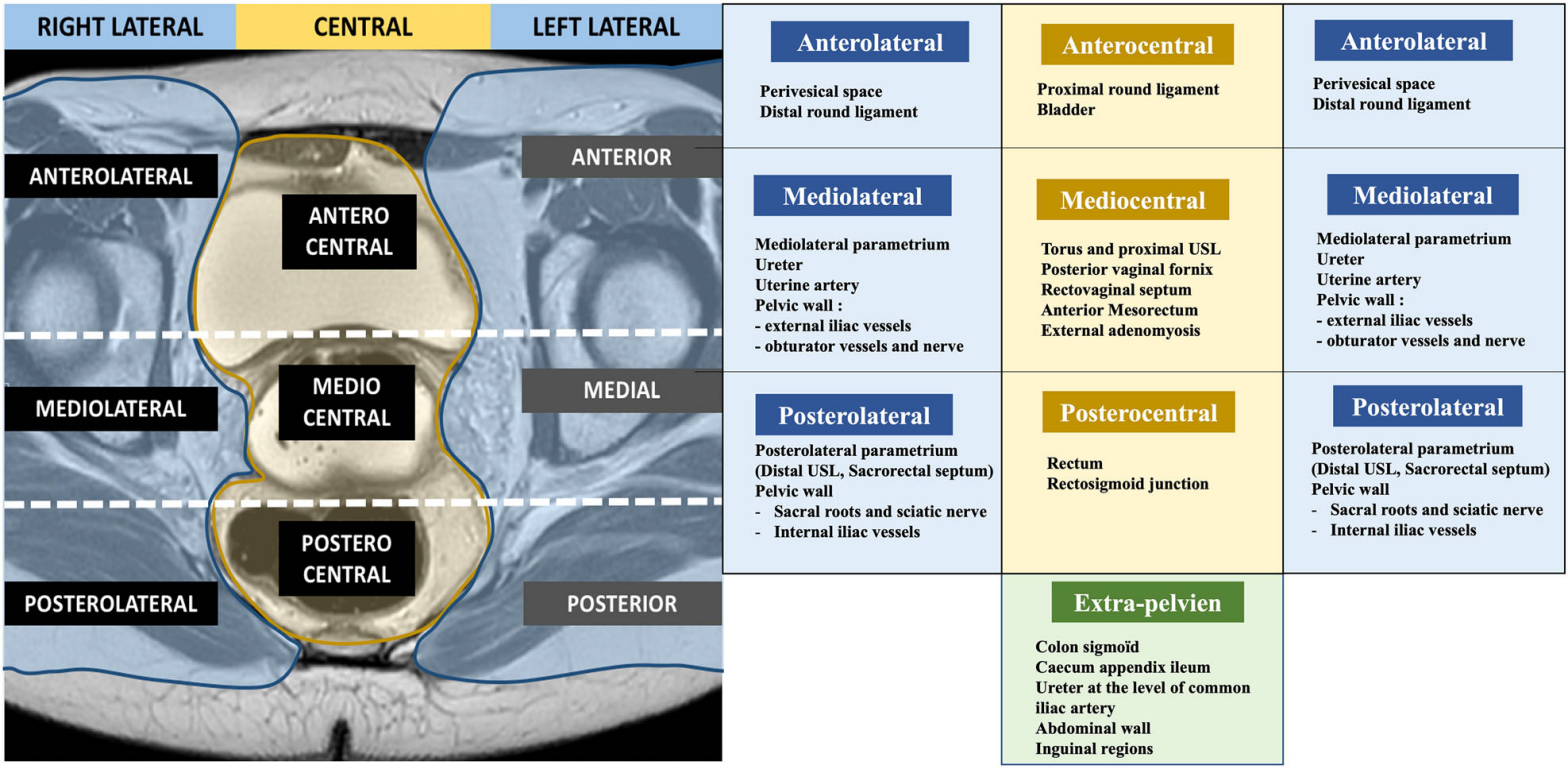
dPEI MR classification. dPEI classification is based on a division of the pelvis into 9 compartments and the description of one extrapelvic compartment. One point was allocated per compartment where any DPE lesion was detected, and an additional point was allocated when an endometriotic lesion involved the pelvic wall in the lateral compartment. One point could be added if a location was found in the vagina or in the trigone (defined as the lower part of bladder base), or if ureteral dilatation was observed [27]. The extent of the disease was defined as follows: mild disease (score ≤ 2); moderate disease (scores 3 and 4), and severe disease (score ≥ 5). Modified from Rousset P, Florin M, Bharwani N et al (2023) Deep-pelvic infiltrating endometriosis: MRI consensus lexicon and compartment-based approach from the ENDOVALIRM group. Diagn Interv Imaging. 104:95–112 Copyright © 2023

In fact, only one radiological score, the deep-pelvic endometriosis index (dPEI) score [5], has been externally validated [9]. In a multicenter study involving over 600 patients, this score is demonstrated to be efficient, simple, and reproducible. It is primarily a quantitative score that accounts for the distribution of DE in nine pelvic quadrants and one extrapelvic quadrant, assigning one point when at least one DE lesion is present. The size of the lesions was not considered, as it is in the Peritoneal Cancer Index, which allocates 1 to 3 points based on the size of the metastatic peritoneal deposits. Given the absence of an established size threshold and inherent inter-observer variability in measurement, a complementary semi-qualitative approach was adopted, proposing the addition of one point for lesions requiring more extensive surgical procedures (involving the vagina, trigone, or ureteral dilation). The extent of the disease is categorized as follows: mild (score ≤ 2), moderate (score 3 and 4), and severe (score ≥ 5). The dPEI score shows a linear increase in operating time and hospital stay corresponding to each score point, better stratification of complication risks, and improved outcomes, such as postoperative voiding dysfunction, as well as a better inter-observer agreement between junior and senior readers. Moreover, the dPEI score provides a straightforward and standardized description of the anatomical locations of DIE (Fig. 1).

Another radiological classification named Endostage was subsequently published [19] with similar goals to those of the dPEI study. This classification includes both deep pelvic locations and adnexal endometriosis. However, this study has many major methodological limitations, such as monocentric retrospective design, absence of any assessment of inter-observer agreement, and no consideration of lateral locations in severe categories.

**Fig. 2:**
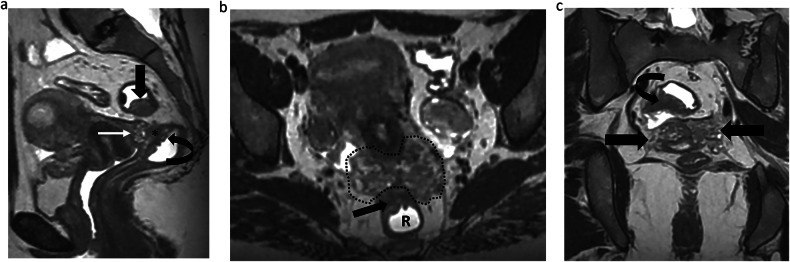
Endometriosis in a 36-year-old woman with deep dyspareunia and chronic constipation. **a** Sagittal T2-weighted image shows a retrocervical nodule (white straight arrow) involving posterior vaginal wall associated with obliteration of the pouch-of-Douglas (*) (mediocentral compartment) and rectal infiltration (curved arrow) (posterocentral compartment). Another nodule affecting the rectosigmoid colon is noted (black straight arrow). **b**, **c** Axial (**b**) and coronal (**c**) T2-weighted MR images show bilateral mediolateral parametrial extension (area inside the dashed line in (**b**) and straight arrows in (**c**), inferring involvement of the inferior hypogastric plexus. Bowel involvement is also noted (straight arrow in **b** and curved arrow in **c**). Thus, this patient is quoted with a dPEI score of 5 (severe disease) with 4 compartments affected (mediocentral, posterocentral, right mediolateral, left mediolateral)

**Fig. 3:**
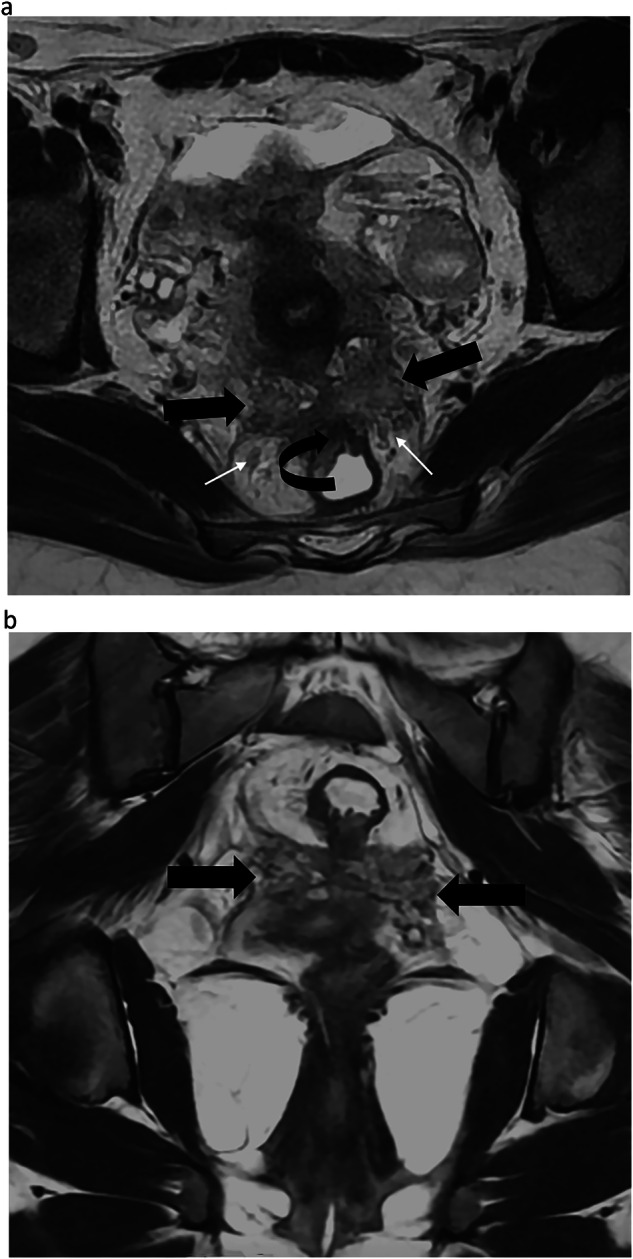
Endometriosis in a 39-year-old woman with deep dyspareunia, chronic constipation, and weak urinary flow. **a** Axial T2-weighted MR image shows a retractile fibrotic mass (straight arrows) involving the torus uterinus, uterosacral ligaments, pouch-of-Douglas (mediocentral compartment), and rectum (curved arrow) (posterocentral compartment), with bilateral mediolateral (dark arrow) and posterolateral parametrium (white arrow). Bilateral involvement of the inferior hypogastric plexus, hypogastric nerves (white arrows), and splanchnic roots is also noted. **b** Coronal T2-weighted MR image shows the parametrial extension of the lesions (arrows). Thus, this patient is quoted with a dPEI score of 6 (severe disease) with 6 compartments affected (mediocentral, posterocentral, right and left mediolateral, right and left posterolateral)

ESUR panelists consider the roles of an MR classification: to improve communication between radiologist and surgeon (100%, 20/20), to improve the communication between the radiologist and the patient (45%, 9/20), to predict operating time if surgery is planned (70%, 14/20), to predict the length of hospital stay after surgery (40%, 8/20), and to predict postoperative complications (70%, 14/20). Moreover, ESUR panelists strongly agree that using an MR classification is useful (19/20 95%). Among the experts that currently use an MR classification in clinical practice (*n* = 13), most of them considered that the most useful classification is the dPEI score (84,6%, 11/13) (Figs. 2 and 3).

**Statement 2:** ESUR panelists strongly agreed with the usefulness of MR classification, with a majority of experts considering the dPEI classification to be the most useful (Grade A).

#### Usefulness of standardized MR report

There is increasing evidence supporting the use of structured reporting in radiology [20, 21]. It is well-recognized that standardized reporting enhances communication between radiologists, referring physicians, and patients, while also benefiting research efforts. Structured reporting ensures that key features are less likely to be overlooked by radiologists and surgeons, provides greater clarity in findings, aids in patient counseling and management, and facilitates more accurate and appropriate data collection for scientific purposes [20]. Additionally, structured reports improve the complementary practices between radiologists and gynecologic surgeons, with the potential to enhance surgical decision-making compared to routine reviews [22].

Detecting endometriosis during laparoscopy can be challenging due to the variable appearance of lesions, the presence of subtle retroperitoneal implants, and the inherent complexities of evaluating pelvic anatomy surgically. Surgeries can also be highly complex and carry significant morbidity, often necessitating multidisciplinary teams, including the assistance of colorectal surgeons and/or urologists. Consequently, a compartmentalized review of pelvic anatomy is seen as a key strength in enhancing diagnostic sensitivity. The significance of a comprehensive compartmental review of pelvic structures by radiologists is highlighted by its critical role in surgical planning. The ESUR expert group strongly agreed with using a structured report (17/20, 85%). Only 11/20 (55%) ESUR panelists admitted to using it in daily practice, but 6/20 answered they will in the future (see ESUR standardized report in the supplementary material).

**Statement 3:** ESUR panelists strongly agreed that a standardized MR report is recommended (Grade B).

#### Usefulness of drawings

Radiology is deeply integrated into the patient care pathway and plays a crucial role in shaping the patient experience. All experts have highlighted the importance and value of clear communication with patients. The traditional models of care, where imaging findings were communicated solely between the radiologist and the referring physician, and management decisions were made without patient input, are no longer viable. The concept of the patient as a passive participant in the decision-making process is increasingly unacceptable [17]. Patient-centered care demands that patients be involved in all major decisions, but this involvement is only meaningful if patients are fully informed about all relevant information, including radiology findings.

“Patient-centered care” is a key dimension of quality care. Patients with confirmed endometriosis often express dissatisfaction with their medical support, and a lack of information is the most significant indicator of dissatisfaction with their overall patient experience [23]. Communication, information, and education have been previously identified as critical areas for improvement in endometriosis clinics, as they are highly valued by patients and are frequently perceived negatively [24]. Beyond procedural accuracy, patients place great importance on receiving information and having clear communication with their radiologist, including explanations of results and personal consultations. However, effective communication with patients can be challenging in busy radiology departments, where the focus is often on efficient and accurate diagnosis [4]. Despite this, optimized communication between patients and radiologists is a vital component of delivering patient-centered and value-based care. Therefore, the availability of the radiologist, as well as the tools they use to explain the diagnosis, is of paramount importance and is integral to the concept of value-based radiology [25].

Furthermore, studies, such as those by Gutzeit et al, report that the opportunity for patients to discuss MRI findings before leaving the department significantly increases their confidence in the radiology service [26]. Good communication with patients, as well as among the healthcare team, has the potential to improve care coordination, enhance safety and outcomes, increase patient satisfaction, and reduce healthcare costs.

In this setting, the role of drawings to illustrate disease mapping is crucial. These drawings significantly aid clinical counseling and, more importantly, surgical planning. They are invaluable for guiding surgeons during complex procedures, making it essential to highlight their importance. This was the main reason for including diagrams in reports.

**Fig. 4:**
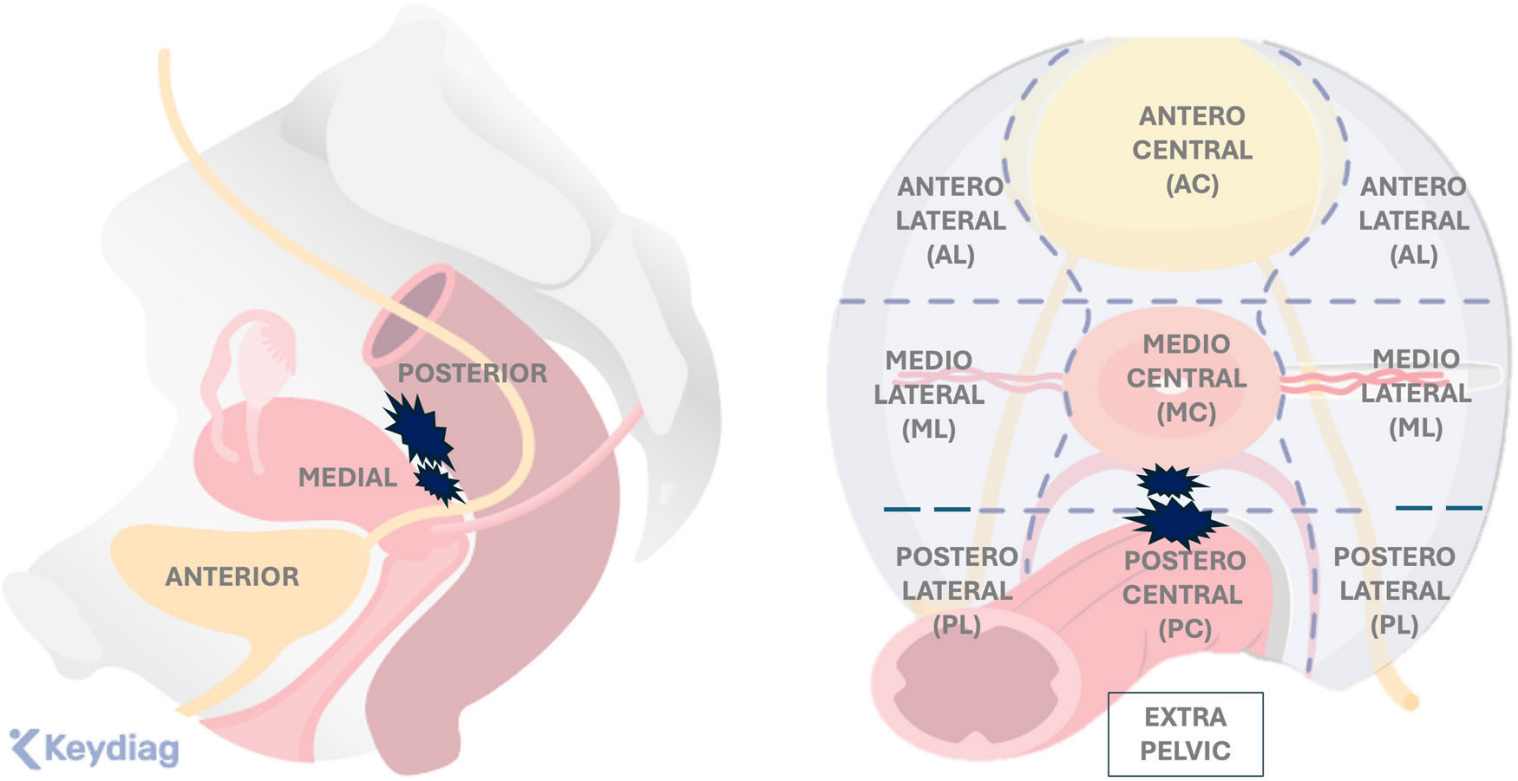
Importance of drawings for improving communication

Among the ESUR expert group, 9/20 experts (45%) use or agreed to use drawings in their report to improve communication with patients (user = 3/20, not yes but will 6/20) (Fig. 4).

**Statement 4:** The use of drawings in an MR report is considered an option, knowing that communication with the patient and surgeon is of paramount importance (Grade D).

In conclusion, the ESUR consensus on endometriosis emphasizes the importance of standardized reporting and MR classifications to enhance communication between radiologists and the multidisciplinary team, as well as between radiologists and their patients. This is crucial in managing a disease where optimized communication is essential for providing patient-centered and value-based care

## Supplementary information

ELECTRONIC SUPPLEMENTARY MATERIAL

## Abbreviations

DE: Deep-pelvic endometriosis
dPEI: deep-Pelvic Endometriosis Index
ESUR: European Society of Urogenital Radiology
MRI: Magnetic resonance imaging
TVUS: Transvaginal ultrasonography
USL: Uterosacral ligament
