# Supplementary material for: ESUR consensus MRI for endometriosis: indications, reporting, and classifications
Source: Eur Radiol. 2025 May 27;35(11):7260–8. doi: 10.1007/s00330-025-11579-0 (PMC12559033; doi:10.1007/s00330-025-11579-0)

ESUR Consensus MRI for Endometriosis: Indications, reporting, and classifications

ELECTRONIC SUPPLEMENTARY MATERIAL

ESUR Standardized Report

CLINICAL INFORMATION
[<>] years old patient, Gravidity [<>] Parity [<>], day [<>] of the cycle, (number of cesarean sections) referred for clinical suspicion of endometriosis: chronic pelvic pain and/or dysmenorrhea (EVA), and/or deep dyspareunia, and/or dysuria / dyschezia during menstruation, and/ or menstrual scapular pain (side) / Infertility
No history of pelvic surgery or pelvic infection.
Hormonal treatment:
Desire for pregnancy.
Referring Physician: Dr. [<>]

TECHNIQUE
Performed on 1.5/3.0Tesla machine
Bowel preparation (Fasting, enema (type of enema), and administration of antispasmodic agents (type))
T2-weighted images of the pelvis were obtained in sagittal plane, axial plane, and coronal plane,
3D Cube/Space T2 weighted images/ Thin sliceT2 weighted images obtained in oblique plane in the axis of the uterosacral ligaments
3DT1 weighted images of the pelvis.
+/- Vaginal opacification
Image quality: satisfactory/degraded by motion artifacts

FINDINGS
The uterus is normal in size and shape, anteverted/retroverted, anteflexed/ retroflexed, displaced to right/left, measuring [<>] x [<>] x [<>]mm
The myometrium displays normal signal intensity. absence/presence of fibroids (description with FIGO type)
Absence/presence of internal adenomyosis: diffuse or focal thickening of junctional zone, with/without cystic foci.

The endometrium is normal measuring [<>] in thickness.
Focal endometrial lesion is detected (description)/No detectable intracavitary anomaly.
The cervix is unremarkable/ abnormal (abnormality description)
C-section scar [<>]

The ovaries are normal in size and show presence of multiple/few small follicles. No adnexal mass lesion is detected/adnexal mass (description)
Right/left hydrosalpinx measured at [<>], with fluid or hemorrhagic signal.

Absence /Presence of free fluid in the pelvis

Adnexal Endometriosis:
Endometriomas/endometriotic implants (side, number, size, central or peripheral, typical or not),
Endometriotic hematosalpinx right/left measured at [<>]
Normal ovarian parenchyma (size)

Superficial Endometriosis:
Implants, adhesions

Deep Endometriosis:
Mid-central compartment: Torus / Proximal USL/ Posterior vaginal fornix/ Rectovaginal septum/ Anterior/ Posterior external adenomyosis measuring [<>]mm

Postero-central compartment: Rectum / rectosigmoid over [<>] mm in length at [<>] cm from the anal margin. This involvement measures [<>]mm in thickness and concerns [<>] degrees of the circumference
Single or multiple

Antero-central compartment: Bladder (dome/base/trigone at [<>] mm from the right/left ureteral meatus)

Lateral compartments:
Over visceral fascia: Right/left mediolateral parametrium / Distal right/left USL/ Right/left posterolateral parametrium/ Anterolateral parametrium
Over parietal fascia: Right/left pelvic sidewall / Ischial notch / Levator muscles / Sacral roots

Extrapelvic compartment: Ileocecal-appendiceal junction / sigmoid colon / Anterior abdominal wall

Complete obliteration of the pouch of Douglas with loculated fluid, highlighting pelvic adhesions

CONCLUSION:

Adnexal endometriosis: Endometrioma (right/left ovarian endometrioma of … mm, hematosalpinx)

Superficial peritoneal endometriosis (implants, adhesions)

Deep pelvic endometriosis involving torus, uterosacral ligaments, vagina, and rectosigmoid over x mm with Douglas' symphysis, médio lateral/postero lateral parametrium, and anterolateral involving vesicouterine pouch, bladder, and round ligaments.
dPEI score XX mild, moderate, severe with [<>] compartments involved

IMAGES AND DRAWINGS

Key Images with arrows

Drawings

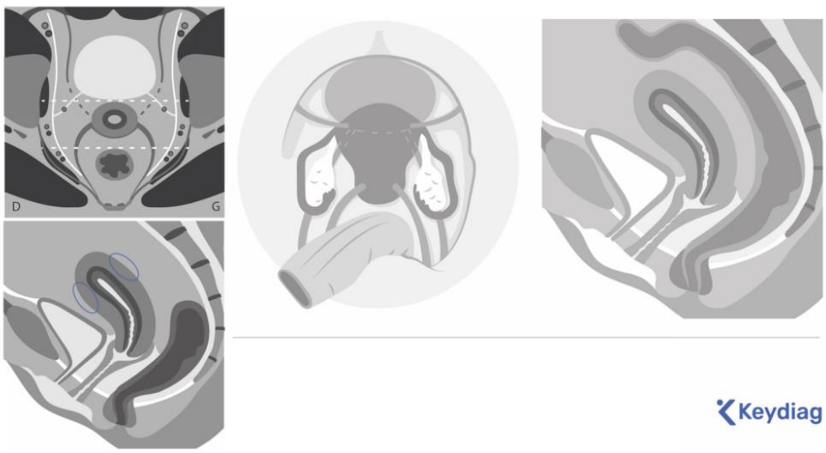

Supplement: Supplementary file 1 — ELECTRONIC SUPPLEMENTARY MATERIAL [file 330_2025_11579_MOESM1_ESM.pdf]
